# Construction of a novel six-gene signature to predict tumour response to induction chemotherapy and overall survival in locoregionally advanced laryngeal and hypopharyngeal carcinoma

**DOI:** 10.1016/j.gendis.2023.05.018

**Published:** 2023-07-16

**Authors:** Chen Tan, Lingwa Wang, Yifan Yang, Shizhi He, George G. Chen, Jason YK. Chan, Michael CF. Tong, C.A. van Hasselt, Wenbin Xu, Ling Feng, Ru Wang, Jugao Fang

**Affiliations:** a Department of Otolaryngology Head and Neck Surgery, Beijing Tongren Hospital, Capital Medical University, Beijing 100730, China; b Key Laboratory of Otolaryngology Head and Neck Surgery (Ministry of Education of China), Beijing Institute of Otolaryngology, Beijing 100730, China; c Department of Otorhinolaryngology, Head and Neck Surgery, The Chinese University of Hong Kong, Prince of Wales Hospital, Hong Kong SAR 999077, China; d Department of Medical Genetics, Institute of Basic Medical Science, Chinese Academy of Medical Science & Peking Union Medical Collage, Beijing 100730, China

Locoregionally advanced laryngeal and hypopharyngeal cancers (LA-LHCs) are traditionally treated with surgery followed by postoperative radiotherapy, which impairs speech and swallowing functions and reduces the quality of life.^1^^,^^2^ The use of induction chemotherapy (IC) as a larynx-preserving approach for LA-LHCs has been verified and refined.^3^ However, the short-term tumor response to IC varies, non-responders usually show poor survival and little benefit.^4^ Therefore, it is crucial to identify IC responders and avoid ineffective treatment. We sought to develop and independently validate a gene-expression signature to predict the efficacy of IC in LA-LHC patients, which would help clinicians select patients who would benefit from IC and provide individual advice for precision treatment, paving the way toward biomarker-driven treatment strategies (Fig. 1).

**Figure 1:**
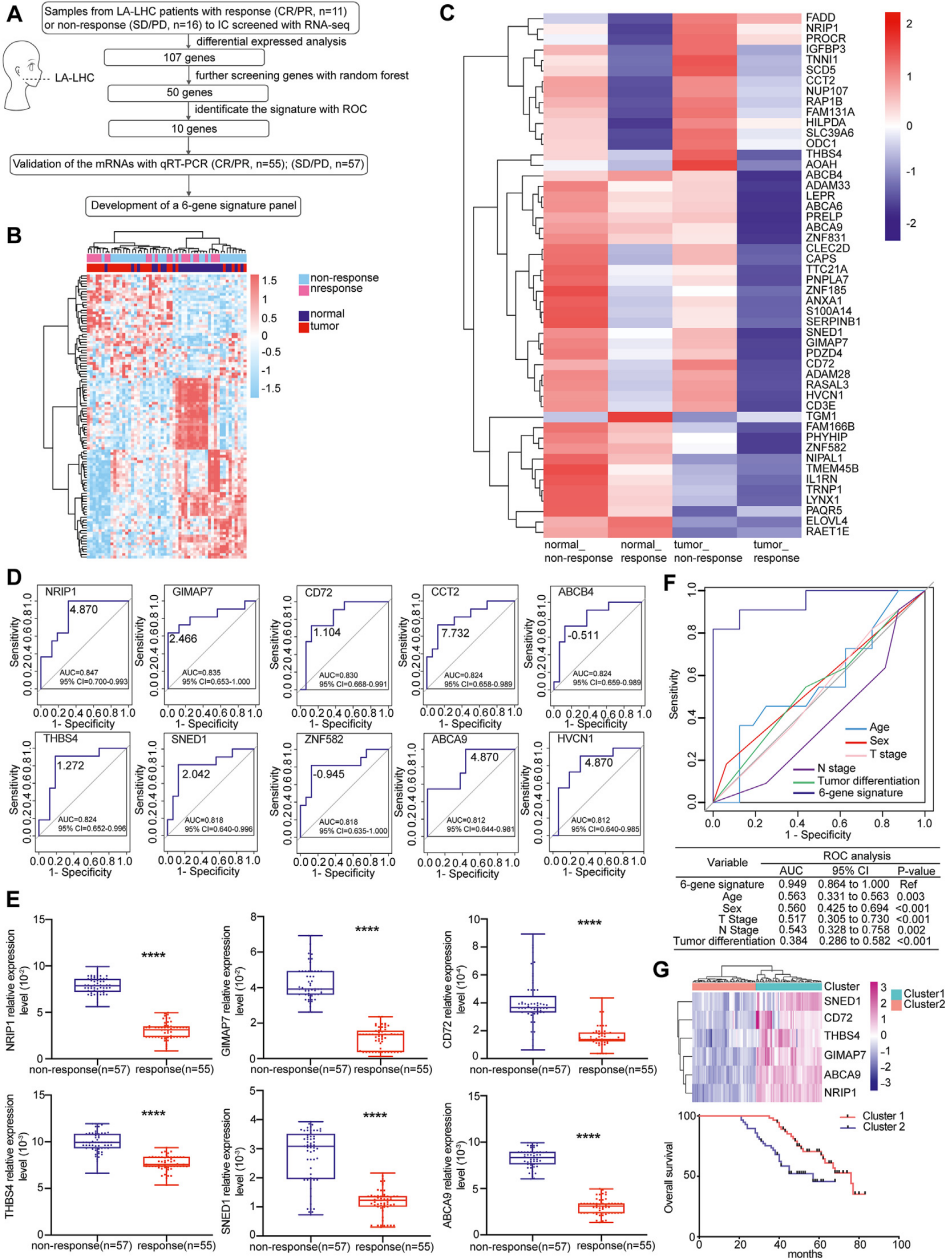
Construction of a novel six-gene signature to predict tumor response to IC and overall survival in locoregionally advanced laryngeal and hypopharyngeal carcinoma. **(A)** Study design. **(B)** Heatmap of 107 selected DEGs, which not only effectively distinguished the tumors from normal tissues, but also distinguished response tumors from non-response tumors. The red and blue points represent up-regulated and down-regulated mRNA expression, respectively. **(C)** Heat map of the 50 genes that not only distinguished between the response and non-response tumors but also distinguished between the tumor tissues and adjacent normal tissues. Red represents an increase in the mRNA expression and blue represents a decrease in the mRNA expression. **(D)** ROC analysis of 10 genes predicting tumor response to IC in LA-LHC patients (AUC >80%). **(E)** Relative expression of six genes validated by qRT-PCR in response and non-response LA-LHC tissues. **(F)** ROC curve analysis of the six-gene signature and clinical variables for the prediction of short-term tumor response to IC. The 95% confidence interval of AUC and *P* value were estimated using the bootstrap method. **(G)** Six genes showed low expression in Cluster 1 and high expression in Cluster 2. Kaplan–Meier analysis showed that overall survival rates were significantly better in Cluster 1 patients than in Cluster 2 patients (*P* < 0.05).

We collected 166 pre-treatment LA-LHC samples, which underwent IC with docetaxel, cisplatin, and fluorouracil. To exclude the influence of individual patient differences, 751 differentially expressed genes (DEGs) were identified between tumor and adjacent normal tissues in each patient (fold change >2.0, *P* < 0.05), including 317 up-regulated and 434 down-regulated genes. To screen IC response-related genes, 636 DEGs were found between response and non-response tumor tissues (fold change >2.0, *P* < 0.05), including 138 up-regulated and 498 down-regulated genes. Finally, 107 DEGs were obtained from the intersection of the two groups, which distinguished tumors from normal tissues, and differentiated between response and non-response tumor tissues.

The 107 DEGs showed distinct transcriptome segregation between response and non-response tumor tissues by principal component analysis. To narrow down the 107 DEGs for further analysis, a random forest model was used to rank these genes in order of importance score from largest to smallest. The top 50 genes were chosen for further analysis showing distinct transcriptome segregation between response and non-response tumor tissues by principal component analysis. Cluster analysis showed that these genes could perfectly distinguish response and non-response samples, which also held true for differentiating between tumor tissues and adjacent normal tissues. Thereafter, the performance of the 50 genes was evaluated to predict the tumor response to IC using receiver operating characteristic (ROC) analysis; among them, 10 genes were chosen of which the area under the curve (AUC) was larger than 80%, and their relative expression was validated by real-time reverse transcription polymerase chain reaction (qRT-PCR) in an independent cohort (57 non-response and 55 response tissues). Among the 10 genes, six genes (*NRIP1*, *GIMAP7*, *CD72*, *THBS4*, *ABCA9*, and *SNED1*) were significantly overexpressed in non-response tissues than in response tissues (*P* < 0.05), exhibiting the same tendency as that in RNA sequencing data. A six-gene signature was constructed to predict the tumor response to IC in LA-LHCs with an AUC of 0.949 by ROC analysis (95% confidence interval = 0.864–1.000). The performance of the six-gene signature in predicting IC tumor response was superior to other clinical parameters (age, sex, T stage, N stage, and tumor differentiation) in LA-LHC patients.

Next, we assessed whether the six-gene signature could be a prognostic factor for LA-LHC patients. Patients were divided into two clusters by K-means clustering according to the expression pattern of the six genes; they were expressed at low and high levels in Cluster 1 and Cluster 2 patients, respectively. Overall survival rates were significantly higher in Cluster 1 than in Cluster 2 patients (*P* < 0.05).

We identified a six-gene signature with high predictive performance for IC efficacy in LA-LHC patients. To our knowledge, this was the first and most comprehensive study to date to demonstrate the prediction accuracy of the six-gene signature in LA-LHC patients undergoing IC. IC efficacy prediction is the main factor in precision treatment decisions for LA-LHCs. In summary, the six-gene signature could screen out IC responders and predict overall survival, avoiding unnecessary IC-related toxicities, expenses, and prolonged waiting periods before treatment.

## Conflict of interests

The authors declare no conflict of interests in this work.

## Funding

The authors were supported by the National Key R&D Program of China (No. 2020YFB1312805).

## Data statement

The data have been deposited in the National Center for Biotechnology Information Gene Expression Omnibus (www. ncbi.nlm.nih.gov/geo/) with the accession number GSE184072.
